# Quality of care and health-related quality of life of climacteric stage women cared for in family medicine clinics in Mexico

**DOI:** 10.1186/1477-7525-8-20

**Published:** 2010-02-10

**Authors:** Svetlana Vladislavovna Doubova Dubova, Sergio Flores-Hernández, Leticia Rodriguez-Aguilar, Ricardo Pérez-Cuevas

**Affiliations:** 1 Unidad de Investigación Epidemiológica y Servicios de Salud Centro Médico Nacional Siglo XXI, Instituto Mexicano del Seguro Social, México DF, México; 2 Coordinación de Investigación en Salud, Centro Médico Nacional Siglo XXI, Instituto Mexicano del Seguro Social, México DF, México

## Abstract

**Objectives:**

1) To design and validate indicators to measure the quality of the process of care that climacteric stage women receive in family medicine clinics (FMC). 2) To assess the quality of care that climacteric stage women receive in FMC. 3) To determine the association between quality of care and health-related quality of life (HR-QoL) among climacteric stage women.

**Methods:**

The study had two phases: I. Design and validation of indicators to measure the quality of care process by using the RAND/UCLA Appropriateness Method. II. Evaluation of the quality of care and its association with HR-QoL through a cross-sectional study conducted in two FMC located in Mexico City that included 410 climacteric stage women. The quality of care was measured by estimating the percentage of recommended care received (PRCR) by climacteric stage women in three process components: health promotion, screening, and treatment. The HR-QoL was measured using the Cervantes scale (0-155). The association between quality of care and HR-QoL was estimated through multiple linear regression analysis.

**Results:**

The lowest mean of PRCR was for the health promotion component (24.1%) and the highest for the treatment component (86.6%). The mean of HR-QoL was 50.1 points. The regression analysis showed that in the treatment component, for every 10 additional points of the PRCR, the global HR-QoL improved 2.8 points on the Cervantes scale (coefficient -0.28, P < 0.0001).

**Conclusion:**

The indicators to measure quality of care for climacteric stage women are applicable and feasible in family medicine settings. There is a positive association between the quality of the treatment component and HR-QoL; this would encourage interventions to improve quality of care for climacteric stage women.

## Introduction

The climacteric stage is the transition from the reproductive to the non-reproductive period during the life of women [1], and comprises 2-8 years before and after menopause [2]. During the climacteric stage, the decline in ovarian hormones and aging contribute to the appearance of climacteric symptoms, decrease in bone mass density, and increase in chronic diseases [2].

This complex scenario may negatively affect the woman's health-related quality of life (HR-QoL) [3] and increases her need for health services [4]. The definition of HR-QoL is as follows: "the perception of a person about his/her physical and psychological health, level of independence and social relationships" [5]. HR-QoL is a proxy for health status, and an outcome variable of epidemiological, clinical, and health systems research studies; it is also an independent predictor for the analysis of the use and cost of health services [6,7].

Measuring the HR-QoL is relevant during the climacteric stage. Hot flashes and sweating can cause anxiety, social isolation, and difficulties at work, which in turn affects HR-QoL [3,8]. Factors such as older age, lack of partner and/or children, unfavorable socioeconomic conditions, low social support, presence of chronic diseases, obesity, and unhealthy lifestyles are associated with low HR-QoL as well [9-11].

Reports from clinical trials have shown that hormone therapy (HT) decreases climacteric symptoms and has a positive effect on HR-QoL [9,12,13]. However, there are no studies aimed at measuring the quality of health care that climacteric stage women receive and its relationship with HR-QoL.

The quality of health care is a multidimensional concept that includes "the degree to which health services for individuals and populations increase the likelihood of desired health outcomes and are consistent with current professional knowledge" [14]. The approach to assess quality should address either individual or population perspectives; in both, it is appropriate to include in the assessment any of the usual three dimensions: structure, process, and outcomes [15].

Process of care is the actual provision and reception of care through interactions between users and providers. At the individual level, measuring the quality of the process of care through indicators is a robust approach [16]. The indicators can measure different components of the process of care, and should be constructed upon standards of care that follow systematic methods based on scientific evidence and/or expert opinion, and should be replicable. The indicators allow valid judgments of the quality of care to be reached and, although they do not provide definitive answers, allow the identification of potential problems during the provision of health care [17].

The growing number of climacteric stage women and the increasing body of knowledge about the complexity of their health needs are raising new requirements for health services.

Health care for climacteric stage women should be comprehensive. This comprises the provision of hormone therapy (HT) when appropriate for climacteric symptoms, and should include counseling about climacteric and menopause, promotion of a healthy lifestyle, and screening, diagnosis, and treatment of chronic diseases. These components must fulfill standards of care that can meet the expectation to achieve a positive effect on the health status and HR-QoL of women.

To build up the evidence on this topic, this study had the following objectives: 1) To design and validate indicators to measure the quality of the process of care that climacteric stage women receive in family medicine clinics. 2) To assess the quality of care that climacteric stage women receive in family medicine clinics. 3) To determine the association between quality of care and health-related quality of life among climacteric stage women.

## Methods

The study was conducted in two phases: I. Design and validation of indicators to measure the quality of care that climacteric stage women receive in family medicine clinics. II. Assessment of the quality of care and of its association with HR-QoL in climacteric stage women.

### Phase I

To design and validate indicators, we used the modified version of the RAND/UCLA Appropriateness Method [18]. This method combines expert opinion and systematic literature review of scientific evidence [19].

The method comprised the following activities:

i) Systematic search and review of the literature to collect scientific evidence regarding the care process activities that climacteric stage women should receive at the family medicine clinic. The databases of Medline, Ovid, Cochrane Library, National Institute for Clinical Excellence, and World Health Organization covering the period 1990-2008 were consulted. The entries for the search were "climacteric" and/or "menopausal" and/or "postmenopausal women," "quality of care indicators" and "guidelines," and "family medicine clinics" or "primary care services."

We identified five systematic reviews, four meta-analyses, and 128 publications that included clinical practice guidelines, clinical trials, and cohort, case-control and cross-sectional studies relevant to answering the scientific question. The criteria of Saslow were used to scrutinize and classify the literature according to the study type and the level of evidence [20].

The systematic literature review allowed the identification of three key components of the process delivered to climacteric stage women: health promotion, screening, and treatment. Within each component, the critical activities to achieve a positive effect on women's health were identified. The research group proposed 16 indicators to evaluate the quality of the process of care

*ii) *An expert panel was integrated by two gynecologists who specialized in climacteric and menopause, two health systems researchers, and two family doctors. All had proven experience in clinical and health system research, and in the development of clinical guidelines/indicators. Each panelist received by e-mail the information about the study objectives, a list of proposed indicators, and the relevant literature. Panelists were asked to validate the indicators by assigning a value from 1 to 9 (1 = definitely not valid and 9 = definitely valid). The classification of the validity of the indicators followed the criteria of Shekelle [21]. The panelists had to use these criteria to individually rate the proposed indicators. To consider an indicator valid, the median panel rating was set to ≥ 7. This decision was in accordance with a published study [21].

After two e-mail rounds of ranking, one vis-à-vis meeting, and a review for coherence and content validity, a final set of 14 indicators was integrated (Table 1).

**Table 1:** Indicators of quality of care that climacteric stage women receive in family medicine clinics

Indicator	Formula
**I. Health promotion**	

1. Counseling about climacteric stage and menopause in the last year	Number of climacteric stage women who received counseling about climacteric stage, menopause and self-care related activities by the family doctor or other health professionals, in the last year/Total number of women in the sample × 100

2. Nutritional counseling in the last year	Number of climacteric stage women who received nutritional counseling by the family doctor or other health professionals, in the last year/Total number of women in the sample × 100

3. Advice on regular leisure time physical activity in the last year	Number of climacteric stage women who received advice on regular leisure time physical activity by the family doctor or other health professionals, in the last year/Total number of women in the sample × 100

4. Smoke cessation counseling in the last year	Number of current smokers climacteric stage women who received smoke cessation counseling by the family doctor or other health professionals, in the last year/Total number of actively smoking women in the sample × 100

**II. Screening**	

1. Deliberate search of climacteric symptoms in the last year	Number of climacteric stage women who were asked by the family doctor about climacteric symptoms in the last year/Total number of women in the sample × 100

2. Screening for overweight and obesity by calculating the body mass index (BMI) in the last year	Number of climacteric stage women who received overweight and obesity screening through the BMI calculation by the family doctor in the last year/Total number of women in the sample × 100

3. Screening for hypertension by measuring the systolic and diastolic blood pressure in the last year	Number of climacteric stage women that received hypertension screening through measuring the systolic and diastolic blood pressure by the family doctor or other health professionals, in the last year/Total number of women in the sample × 100

4. Screening for diabetes by measuring fasting plasma glucose in the last year	Number of climacteric stage women who received diabetes screening through fasting plasma glucose measurement, in the last year/Total number of women in the sample × 100

5. Screening for breast cancer through mammography in the last 2 years	Number of climacteric stage women who received breast cancer screening through mammography, in the last 2 years/Total number of women in the sample × 100

6. Screening for cervical cancer through Pap test in the last 3 years in women without a history of total hysterectomy	Number of climacteric stage women without a history of total hysterectomy for benign disease who received cervical cancer screening through Pap test, in the last 3 years/Total number of women in the sample without a history of total hysterectomy × 100

**III. Treatment**	

1. Appropriate indication of oral hormone therapy (HT)	a) Number of women with moderate or severe vasomotor symptoms 7/day ≥ (at the time of the interview or the time to start oral HT) and without HT contraindications, who receive oral HT

	b) Number of women with moderate or severe vasomotor symptoms <7/day(at the time of the interview or the time to start oral HT), or with mild symptoms or without vasomotor symptoms who do not receive oral HT/Total number of women in the sample × 100

2. Appropriate indication of vaginal HT	a) Number of women with moderate or severe vaginal atrophy symptoms and without oral HT who receive vaginal HT

	b) the number of women without moderate to severe vaginal atrophy symptoms or with oral HT who do not receive vaginal HT/Total number of women in the sample × 100

3. Appropriate prescription of oral HT	Number of women receiving oral HT prescription appropriately according to the drug scheme, dose, schedule and duration of the treatment/Total number of women in the sample receiving oral HT × 100

4. Information on risks and benefits of oral HT	Number of women who were prescribed oral HT and who received information about its purpose, benefits and risks/Total number of women in the sample receiving oral HT × 100

### Phase II

From November 2008 to March 2009, we conducted a cross-sectional study in two Instituto Mexicano del Seguro Social (IMSS) family medicine clinics (FMC) located in Mexico City. The FMC were randomly selected from the list of existing FMC in Mexico City. One clinic was in the south of the city and the other in the north. Both clinics had similar characteristics, such as the number of examining rooms and people covered.

The IMSS is a social security system for workers in the formal market; ~48 million Mexicans are affiliated with this institution [22].

The study population was women in climacteric stage aged 45-59 years attending the FMC. To identify these women we used the definition of the "Clinical Practice Guideline on the Menopause and Postmenopause" [2]; also we took into account that the mean age in which the menopause occurs among Mexican women is 48 years [23]. Besides the age interval, we also asked postmenopausal candidates the date of the last menstrual period and we only included participants who had their last period no longer than eight years ago. Other inclusion criteria were: at least three visits to the family doctor in the last year; not suffering from type 2 diabetes, hypertension, depression, and/or cancer; being with a stable life partner and agreeing to participate in the study by signing the informed consent.

### Study variables

The dependent variable was HR-QoL, and this was measured with the Cervantes scale [24]. This scale is a specific HR-QoL instrument for menopausal women. The scale has 31 questions and covers four domains: menopause and health, psychological domain, sexuality, and couple relationship. The highest value for the global score is 155 points, which means low HR-QoL, and the lowest value is 0, which means high HR-QoL.

The independent variable was quality of care, which was measured by ascertaining the percentage of recommended care received [25]. This was estimated for each care process component: health promotion, screening, and treatment (Table 1). It was obtained by calculating a simple proportion, with the sum of indicators that women received as the numerator and the total number of the recommended indicators as the denominator.

The covariates were:

a) Women's general characteristics: Age, schooling, and employment status, which included whether she was involved in paid work.

b) Lifestyle: Healthy diet [26-28] which included the daily consumption of fruits, vegetables, and dairy products, and non-consumption of carbonated beverages; leisure time physical activity (PA) [29] where regular was defined as moderate intensity if done for ≥ 30 minutes/day ≥ 5 days/week or vigorous intensity if done three times a week with a duration of 20 minutes per session, irregular was defined as carrying out less than regular PA, or inactivity. Smoking status was focused on current smokers and we registered the number of cigarettes actually smoked per day among those that answered positively. Alcohol consumption was initially classified as non-drinkers (never drink alcohol), occasional drinkers (drink rarely or less than once a week), moderate drinkers (from 1 to 14 drinks per week) and heavy drinkers (more than 14 drinks per week) [30]. It has been reported that moderate alcohol consumption has a positive association with HR-QoL in middle aged women [31]; therefore, we combined non-drinkers and occasional drinkers in a single group and presented the data for moderate alcohol consumption only.

c) Nutritional status was measured by body mass index (BMI) and classified into groups of normal weight (BMI of 18.5-24.9 kg/m^2^), overweight (BMI of 25.0 to 29.9 kg/m^2^), or obese (BMI ≥ 30.0 kg/m^2^).

d) Social support (SS) was measured by applying the DUKE-UNC-11 questionnaire [32]. This questionnaire evaluates confidential SS (possibility of having people to communicate with) with a minimum score of 7 points (low confidential SS) and a maximum score of 35 points (high confidential SS); and affective SS (demonstration of love, affection, and empathy) with a minimum score of 4 (low affective SS) and a maximum score of 20 points (high affective SS).

e) Medical and reproductive history: Presence of chronic diseases, number of pregnancies and living children, and menopause (one year after the last menstrual period). Type of menopause was classified as natural or surgical, age at onset of menopause; time elapsed since menopause, presence and type of climacteric symptoms, and number of visits with the family doctor in the last year. The severity of vasomotor symptoms and vaginal atrophy symptoms was classified using the criteria proposed by the Department of Health and Human Services Food and Drug Administration [33], which are based upon women's self-report, and define the symptoms as mild, moderate, or severe.

f) Satisfaction with care received at the FMC was measured with the general question of how satisfied are you with the care you have received at the FMC? The possible answers were very satisfied, satisfied, neither satisfied nor unsatisfied, unsatisfied, and very unsatisfied.

### Sample size

We estimated a sample size of 400 women to evaluate the possible association between HR-QoL and quality of care. The sample size was estimated by using the formula to test the mean of a normal distribution [34]. A mean decrease of at least 5 points on the global Cervantes scale score per 10% increase in the quality of care received was considered to be clinically relevant. The assumptions included a mean global HR-QoL score of 51.75 points (standard deviation of 23.1 points) [24], α error = 0.05, 80% power, and 10% of possible non-respondents (this means that a respondent answered less than 80% of the questionnaire).

### Study description

In each FMC, the nurse identified candidates in the waiting room, explained the purpose of the study and of the interview, and asked for her signed informed consent. If the candidate agreed to participate, the nurse performed the interview. The questionnaires used during the interview were the Cervantes scale, the DUKE-UNC-11 questionnaire, and a structured questionnaire to collect general information and data to measure quality of care.

All questionnaires, including the Cervantes scale, were pretested in 25 women in climacteric stage regarding their understanding of the questions. The supervisory nurse and/or one of the researchers (SVD) reviewed the previous year's clinical notes in the electronic medical record to verify the care that each woman received.

The project was approved by the National Research and Ethics Committee of the IMSS (number 2008-785-014).

### Statistical analysis

The descriptive analysis consisted of obtaining measures of central tendency and dispersion for quantitative variables; in the case of categorical variables, absolute and relative frequencies were obtained.

For the descriptive analysis of the HR-QoL, the mean and standard deviation (SD) of global and particular domain scores were obtained. We also categorized HR-QoL global score and domain scores into: 1) low HR-QoL, severe problem level (+2SD); 2) moderately low HR-QoL, high problem level (+1SD and +2SD); 3) regular HR-QoL, low-medium problem level (+1SD and -1SD); and 4) high HR-QoL, without problems (-1SD) [23]

The association between global HR-QoL score and the percentage of recommended care received for each component of care (health promotion, screening and treatment), as well as for each of the covariates was evaluated through the Spearman correlation test.

To determine the magnitude of the adjusted association between HR-QoL global score and each component of quality of care, we used multiple linear regression analysis. The model included conceptually relevant variables (schooling, confidential and affective support, leisure time physical activity, healthy diet, presence of chronic disease, body mass index, menopause, and satisfaction with health care) that resulted in p ≤ 0.20 in the bivariate statistical analysis. The method used for modeling was backwards. The covariate presence/absence of menopause was included as an adjustment variable and it was not statistically significant, although given its clinical importance it was maintained during the modeling process. It was also tested if menopause influenced the association of interest (relationship between HR-QoL global score and each component of quality of care). The analysis tested the interactions between each component of quality of care and menopause; such interactions were not statistically significant and were not included in the final model.

Once the final model was obtained, the error terms were generated, the assumptions of linearity, normality, and equal variance were tested, and the goodness-of-fit of the regression line was confirmed.

The analysis was performed with the Stata 8.0 statistical software (Stata 8.0, Stata Corp; College Station, TX).

## Results

A total of 424 women met the inclusion criteria, of which 2% refused to participate due to lack of time to answer the interview questions. Of the 416 women interviewed, 6 (1.4%) were excluded because they had no medical notes of consultations during the last year in their electronic medical records. The final analysis included 410 women.

### General characteristics, lifestyle, nutritional status, and social support (Table 2)

**Table 2:** General characteristics, lifestyle, nutritional status, and social support

Variables	n = 410 n (%)
**I. General characteristics**	
Years of age, median (minimum- maximum)	49 (45-59)
Years of schooling, median (minimum- maximum)	8 (0-20)
Paid work	144 (35.1)
**II. Lifestyle**	
Healthy diet	85 (20.7)
Leisure time physical activity	
Regular	102 (24.9)
Irregular	67 (16.3)
Inactivity	241 (58.8)
Current smokers	75 (18.3)
Number of cigarettes per day, median (minimum- maximum)	3 (1-15)
Moderate alcohol intake	8 (2.0)
**III. Nutritional status**	
Body mass index, kg/m^2^, mean ± SD	29.1 ± 4.3
Normal weight	70 (17.1)
Overweight	189 (46.1)
Obesity	151 (36.8)
**IV. Social support**	
Confidential, mean ± SD	23.1 ± 6.5
Affective, mean ± SD	15.5 ± 3.7

The median age was 49 years, and the median schooling was at secondary level. Of the respondents, 64.9% were devoted to home and had no paid work.

As for lifestyle, the results show that most of women had an unhealthy lifestyle; one in five women reported having a healthy diet, and one in four reported regular physical activity. Only 6.7% had both a healthy diet and regular leisure time physical activity; 18.3% were current smokers, they smoked a median of three cigarettes per day. Most of interviewees were non-drinkers or occasional drinkers, only 2% reported moderate consumption. It was noted that a high proportion of participants were overweight or obese.

On average, the interviewees received moderate social support. The mean score for confidential support was 23.1 on a scale of 7 to 35 points and the mean score for emotional support was 15.5 points on a scale of 4 to 20 points.

### Medical and reproductive history, and climacteric symptoms (Table 3)

**Table 3:** Medical and reproductive history, climacteric symptoms, and number of consultations with family doctor

Variable	n = 410 n (%)
**I. Medical and reproductive history**	
Presence of chronic disease	202 (49.3)
Number of pregnancies, median (min- max)	3 (1-9)
Number of living children, median (min- max)	3 (0-9)
Menopause	224 (54.6)
**Type of menopause**	n = 224
Natural	162 (72.3)
Surgical	62 (27.7)
**Age in which menopause happened**, median (min- max)	n = 224
Natural	49 (38-56)
Surgical	45.5 (38-53)
Time elapsed after menopause, years	3 (0-8)
**II. Climacteric symptoms**	**n = 410**
Hot flashes	205 (50.0)
Sweating	181 (44.1)
Insomnia	169(41.2)
Dysuria	91 (22.2)
Vaginal dryness	161 (39.3)
	n = 186
Changes in the menstrual cycle in pre-menopause women	98 (52.6)
	n = 352
Dyspareunia and/or vaginal bleeding in sexual active women	101 (28.7)
**Severity and frequency of vasomotor symptoms**	**n = 410**
Absence	192 (46.8)
Mild	46 (11.2)
Moderate	
< 7/day	129 (31.5)
≥ 7/day	2 (0.5)
Severe	
< 7/day	32 (7.8)
≥ 7/day	9 (2.2)
**Severity of vaginal atrophy symptoms**	**n = 410**
Absence	219 (53.4)
Mild	101 (24.6)
Moderate	40 (9.8)
Severe	50 (12.2)
**III. Number of consultations with a family doctor during the last year**, median (min- max)	6 (3-20)

Half of participating women suffered from one chronic condition, mainly musculoskeletal system diseases (41.9%), and nutritional and metabolic disorders such as dyslipidemia (19.0%).

As for reproductive history, the median number of pregnancies and living children was three. Half of the participants were menopausal, of which most had natural menopause. The median age at natural menopause was 49 years and at surgical menopause was 45.5 years; the median time elapsed since menopause was three years. The most frequent climacteric symptoms were changes in the menstrual cycle, hot flashes, sweating, and dyspareunia; very few reported having moderate or severe vasomotor symptoms, with a frequency of 7 or more times a day; 22% had moderate to severe symptoms of vaginal atrophy. Women attended a median of six visits to their family doctors in the last year.

### Quality of care and satisfaction with care (Table 4)

**Table 4:** Quality of care^† ^and satisfaction with care

Variable	n = 410
**I. Health promotion**	n (%)
1. Counseling about climacteric and menopause	52 (12.7)
2. Nutritional counseling	59 (14.4)
3. Advice on regular leisure time physical activity	178 (43.4)
4. Smoke cessation counseling in current smokers	n = 75 23 (30.7)
	
**II. Screening**	**n = 410**
1. Deliberate search of climacteric symptoms	155 (37.8)
2. Screening for overweight/obesity	14 (3.4)
3. Screening for hypertension	407 (99.3)
4. Screening for diabetes	362 (88.3)
5. Screening for breast cancer	173 (42.2)
6. Screening for cervical cancer in women without total hysterectomy	n = 347 319 (91.9)
	
**III. Treatment**	**n = 410**
1. Appropriate indication of oral HT	388 (94.6)
a) Women with moderate or severe vasomotor symptoms ≥ 7/day (at the time of the interview or the time to start oral HT) and without contraindications for HT	n = 37
oral HT indicated appropriately	20 (54.0)
oral HT not indicated	17 (46.0)
b) Women with moderate or severe vasomotor symptoms <7/day (at the time of the interview or the time to start oral HT), with mild symptoms or without vasomotor symptoms	n = 373
oral HT indicated	5 (1.4)
oral HT not indicated appropriately	368 (98.6)
2. Appropriate indication of vaginal HT	332 (81.0)
a) women with moderate or severe vaginal atrophy symptoms and without oral HT	n = 87
vaginal HT indicated appropriately	9 (10.3)
vaginal HT not indicated	78 (89.7)
b) women without moderate to severe vaginal atrophy symptoms or with oral HT	n = 323
vaginal HT indicated	0 (0.0)
vaginal HT not indicated appropriately	323 (100.0)
Women receiving oral HT	n = 31
3. Appropriate prescription of oral HT	16 (51.6)
4. Information on risks and benefits of oral HT	12 (38.7)
	**n = 410**
**Percentage of recommended care received**	% mean ± SD
Health promotion	24.1 ± 28.1
Screening	59.5 ± 16.8
Treatment	86.6 ± 22.9
**Satisfaction with care received at the FMC**	n (%)
Very satisfied	66 (16.1)
Satisfied	200 (48.8)
Neither satisfied nor unsatisfied	90 (22.0)
Unsatisfied	41(10.0)
Very unsatisfied	13 (3.2)

^† ^For complete information about the formula of each quality of care indicator, please see Table 1.

The quality of care was assessed in three domains: health promotion, screening, and treatment. Regarding health promotion, a low percentage of participants had received counseling about climacteric and menopause, nutrition, leisure time physical activity, and smoking cessation.

The screening component showed important limitations in several components. The family doctor asked about climacteric symptoms in 37.8% of participants; ascertainment of overweight and obesity were registered in 3.4%, and screening for breast cancer in 42.2%. Hypertension, diabetes, and cervical cancer screening tests were performed in most women (99.3%, 88.3%, and 91.9%, respectively).

The treatment component indicated that most women had appropriate indication of vaginal and oral HT (94.6% and 81.0%, respectively). While 51.6% of 31 women receiving oral HT had an appropriate prescription in terms of scheme, dose, and time schedule, 38.7% had received information about the risks and benefits of HT.

The health promotion component had the lowest mean percentage of recommended care (24.1%), while the treatment component had the highest (86.6%). Most of the interviewed women (64.9%) reported being satisfied with the care received at the FMC.

### Health-related quality of life (Figure 1)

**Figure 1:**
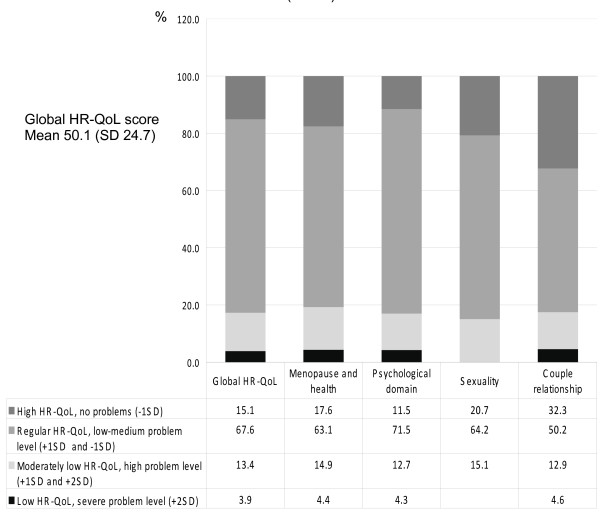
**Health-related quality of life measured by Cervantes scale (n = 410)**.

Women rated their global HR-QoL as follows: high HR-QoL, 15.1%; regular HR-QoL, 67.6%; moderately low HR-QoL, 13.4%; and low HR-QoL, 3.9%. The mean global HR-QoL score was 50.1 points (SD 24.7). The analysis within the domains shows that more women in the couple relationship domain reported high HR-QoL (32.3%) compared with the other domains; in the sexual domain, nobody reported low HR-QoL.

### Relationship between health-related quality of life and quality of care (Table 5)

**Table 5:** Relationship between health-related quality of life^† ^and quality of care

	Coefficient	Confidence intervals at 95%	P value
Percentage of recommended care received:			
Health promotion	-0.01	-0.09; 0.06	0.722
Screening	-0.09	-0.21; 0.04	0.158
Treatment	-0.28	-0.37; -0.19	0.000
Schooling	-1.40	-1.98; -0.83	0.000
Leisure time physical activity			
Regular	-9.83	-14.92; -4.76	0.000
Irregular	-6.01	-12.04; 0.02	0.051
Confidential support	-0.46	-0.85; -0.07	0.022
Affective support	-1.23	-1.93; -0.54	0.001
Body mass index	0.54	0.06; 1.02	0.027
Absence of menopause	-3.08	-7.28; 1.05	0.148
Satisfaction with health care in the FMC	-3.72	-5.88; -1.57	0.001

^† ^Health-related quality of life measured with the Cervantes scale, where low scores indicate better HR-QoL.

The quality of treatment was significantly associated with a better rating of global HR-QoL after adjusting for other variables. The higher mean percentage of recommended care received in the treatment component reduced the mean global HR-QoL score on the Cervantes scale (coefficient -0.28, P < 0.0001). This means that for each 10 percentage points more of recommended care received in the treatment component, the rate of HR-QoL improved by 2.8 points on the Cervantes scale. The association between HR-QoL and the health promotion and screening components was not statistically significant.

## Discussion

The health of women in the climacteric stage is a complex matter that requires further attention from health services [8]. Some of the health problems that a climacteric woman suffers can be prevented or timely diagnosed, thus allowing the control or mitigation of the potential consequences. Promoting high quality care based on scientific evidence is critical; this helps women to age in better health.

We designed and validated 14 indicators addressing health promotion, screening, and treatment to assess the quality of the process of care that climacteric stage women receive in family medicine clinics. The indicators should be feasible, available, and continuous. The information for the present study came from two sources: interviews with climacteric stage women and medical records. Combining both sources provided information that is more reliable but increased the cost of data collection since trained personnel was required. In practice, these indicators allowed the analysis of the quality of care process with a swift and replicable methodology.

The evaluation of the quality of care pointed out flaws in the processes of health promotion, screening, and treatment. This finding is similar to the results of other studies reporting that users receive only about half of the recommended actions [35,36].

Health promotion interventions for climacteric stage women include motivation to quit smoking, to follow a healthy diet, and to do regular leisure time physical activity. Carrying out these activities improves their health status, reduces mortality due to chronic diseases, and maintains bone mineral density and muscle strength [26-28,37,38]. Counseling about climacteric stage increases women's knowledge about it and receptiveness about self-care. Informed women can cope better with the physiological and emotional changes that occur at this stage and improve their lifestyle [39,40]. In our study, the evaluation of health promotion revealed serious limitations. Only one out of every ten women followed a healthy diet and did regular leisure time physical activity. Most of those with unhealthy lifestyles had not received information to improve it.

In family medicine clinics, all members of the health team should perform health promotion activities: medical doctors, nurses, social workers, nutritionists, etc. Ideally, these activities should be complementary, and the health team members should reinforce them continually. Previous studies performed at IMSS have reported that health promotion is inadequate and requires substantial improvements [41].

Screening of diseases allows timely diagnosis and treatment, thus increasing the probability of better health outcomes. The present study showed that women underwent only half of the recommended screening activities. Screening for overweight/obesity was poor, despite it being easy to perform and the high prevalence of obesity among Mexican women [42]. This finding suggests the need to encourage health services to improve the screening activities and to educate women in this age group to increase the informed demand for preventive care.

During the last years, the appropriate indication and prescription of hormone therapy have been debated [43]. Evidence-based clinical guidelines are available for managing climacteric women. These guides provide recommendations for the indication and appropriate use of hormone therapy, while reducing the risk of adverse events. In our study, we found that the treatment component was close to the current recommendations, but only half of the women were receiving appropriate prescription of oral HT.

The information/education of women about the purpose, benefits, and potential adverse events of HT contributes to increased adherence to HT and the timely identification of the adverse events. The flaws of the treatment component, such as inappropriate prescription and poor information, stress the need to update the medical staff and to evaluate the quality of care in a continuous way. In addition, it is advisable to search for feasible alternatives to motivate providers to deliver high-quality care. The use of incentives, either in kind or monetary, is a viable approach. In addition, the definition of standards is necessary as this allows the quality to be evaluated in a reliable way; our data would help in defining the standards of care at the local level. The implementation of standards of care and evaluation activities should be tailored to the characteristics of the services that are being evaluated.

In our study, we used the Cervantes scale to measure HR-QoL because this scale comprehensively addresses the main domains of the health of women in climacteric stage. The questionnaire was applied as rigorously as possible, assuring that all participants understood and answered all the questions. In our sample, the mean value for the global HR-QoL score was similar to those reported for Spanish women [24]. It is noticeable that no woman reported severe problems in the sexuality domain. It is possible that due to sensitive nature of the subject, they were not completely open to answer such question. In another study in Spain that used the Cervantes Scale, it was observed that the answers of women in the sexuality domain were different when they were interviewed directly, than when they answered anonymously the questionnaire. In the open interview they minimized the problems in the sexual domain [44]. In our study, all participants answered to an interviewer. Our findings allow the assumption that the Cervantes scale can be applied to Mexican women but should be validated within the Mexican context.

There is an ongoing trend toward ascertaining the relationship between quality of care and health outcomes [45,46]. The present study found that the treatment component was associated with better global HR-QoL. This can be due to the effect of the drugs on reducing the climacteric symptoms, thus resulting on improving HR-QoL in the short term; in contrast, the positive effect of the health promotion and screening components would happen in the long term. It is possible that the relationship between these components and HR-QoL is not straightforward. A number of variables intervene, such as changes in lifestyle, women's endowment, etc.

This cross-sectional study has several limitations. It is possible that the evaluation of the quality of care is limited because, in Mexico, the women who use health services often have a chronic illness and require specific attention. In this study, the quality of care assessment was limited to the climacteric stage and did not assess the process of care for other health problems. The study evaluated the quality of care only in IMSS-affiliated women; this affects its external validity. It would be pertinent to consider the applicability of the indicators in other health care institutions. This is reasonable because quality measurements should consider the local conditions. In addition, the probability of misclassifying the indicators for evaluating the appropriate oral and vaginal HT indication exists; each indicator combines two parts 1) appropriate indication for women who need this therapy and 2) no indication for women who do not need it. Because most of the women in the sample do not need to receive either oral or vaginal HT, the results reflect the proper "no indication" more, so the association between HR-QoL and quality of care in the treatment component was probably overestimated. Also, to evaluate the HR-QoL we used the Cervantes scale, which has questions about the couple relationship, which in turn implies that the woman should have a partner. This could represent a limitation in the generalizability of the study, given that it has been reported in Mexico that about 21% of women of this age do not have a partner [41].

In conclusion, the indicators developed to measure the quality of care process for climacteric stage women are applicable and feasible. Its application in this study showed that health care in this population is limited in the health promotion, screening, and treatment components. There is a positive association between the quality of treatment and HR-QoL, which can encourage the development of interventions aimed at improving the performance of health services. It is advisable to consider the possibility of designing future interventions with a holistic approach toward improving the quality of care for women at this stage.

## Competing interests

The authors declare that they have no competing interests.

## Authors' contributions

SVD and RPC contributed in conceptualizing the research, data analysis and writing the paper. SFH contributed in the data analysis. LRA contributed in supervising the fieldwork. All authors participated in the interpretation of data, read, and approved the final version of this manuscript.
